# Uncoupling TORC2 from AGC kinases inhibits tumour growth

**DOI:** 10.18632/oncotarget.20086

**Published:** 2017-08-09

**Authors:** Angus J.M. Cameron, Selvaraju Veeriah, Jacqueline J.T. Marshall, Elizabeth R. Murray, Banafshé Larijani, Peter J. Parker

**Affiliations:** ^1^ Kinase Biology Laboratory, Barts Cancer Institute, Queen Mary University of London, John Vane Science Centre, Charterhouse Square, London, UK; ^2^ Translational Cancer Therapeutics Laboratory, Paul O’Gorman Building, University College London Cancer Institute, London, United Kingdom; ^3^ Protein Phosphorylation Laboratory, Francis Crick Institute, London, UK; ^4^ Cell Biophysics Laboratory, Ikerbasque Basque Foundation for Science, Research Centre for Experimental Marine Biology and Biotechnology (PiE) & Biofísika Instituto (UPV/EHU, CSIC), University of the Basque Country, Areatza Hiribidea, Plentzia, Spain; ^5^ Division of Cancer Studies, King’s College London, New Hunts House, Guy’s Campus, London, UK

**Keywords:** mTORC2, CRIM, Akt, xenograft, Sin1

## Abstract

Mammalian target of rapamycin (mTOR) is a central regulator of growth and metabolism. mTOR resides in two distinct multi-protein complexes – mTORC1 and mTORC2 – with distinct upstream regulators and downstream targets. While it is possible to specifically inhibit mTORC1 with rapamycin, or inhibit both mTOR complexes together with ATP pocket directed mTOR kinase inhibitors, it is not possible to assess the specific roles for mTORC2 pharmacologically. To overcome this, we have developed a novel, inducible, dominant negative system for disrupting substrate recruitment to mTORC2. Previously we identified the mTORC2 specific subunit Sin1 as a direct binding partner for AGC kinases Akt and PKC. Sin1 mutants, which retain the ability to bind Rictor and mTOR, but fail to recruit their AGC client kinases, inhibit AKT and PKC priming and block cell growth. In this study, we demonstrate that uncoupling mTORC2 from AGC kinases in DLD1 colon cancer cells inhibits Akt activation and blocks tumour growth *in vivo*. Further we demonstrate, using time resolved two-site amplified FRET (A-FRET) analysis of xenograft tumours, that inhibition of tumour growth correlates with the degree of mTORC2 uncoupling from its downstream targets, as demonstrated for Akt. These data add weight to the body of evidence that mTORC2 represents a pharmacological target in cancer independently of mTORC1.

## INTRODUCTION

The mammalian target of rapamycin (mTOR) is a key regulator of eukaryotic cell growth and represents a major drug target in numerous cancers [1-3]. There are two evolutionary conserved branches to mTOR signalling mediated by two multi-protein complexes, mTORC1 and mTORC2, with distinct roles in cell growth control [4]. While these complexes share many subunits, they are defined by unique protein components, including raptor for mTORC1 or Sin1 and Rictor for mTORC2. These unique subunits confer distinct regulation, localisation and substrate specificities for the two complexes. Structural and biochemical studies are now delineating the precise mechanisms governing upstream regulation and downstream target recruitment of the two complexes

Among the key targets for the mTOR complexes are AGC family kinases, including Akt, p70S6 kinase, SGK and PKC. AGC kinase activity is typically regulated by PDK1 mediated phosphorylation of the generic kinase domain activation loop and mTOR mediated phosphorylation of conserved hydrophobic- and turn-motifs within a C-terminal kinase domain extension. These phosphorylations cooperate to fully activate the AGC kinase domain [5]. mTORC1 and mTORC2 target distinct AGC family members. Thus, mTORC1 phosphorylates p70S6K to regulate protein synthesis, while mTORC2 targets Akt, PKC and SGK downstream of various growth factor stimuli to regulate diverse cellular functions [6-11].

The emergence and early promise of mTOR inhibitors has provided evidence that targeting of mTORC1 with rapalogues, or both mTORC1 and mTORC2 with active site directed inhibitors, may be beneficial therapeutically in a number of malignancies. Dose limiting toxicities [12] and potential caveats in the targeting of mTORC1 under nutrient deprived conditions [13, 14] have also made the development of specific mTORC2 directed therapies an attractive proposition. A number of lines of evidence suggest mTORC2 targeting alone might be beneficial. Prostate specific deletion of Rictor was found to supress PTEN driven tumourigenesis downstream of Akt in mice and mTORC2 mediates Akt dependent chemotherapy resistance in PTEN null glioma patients [15], indicating the importance of mTORC2 as a PI3kinase effector pathway. mTORC2 can also regulate cancer metabolism, survival under hypoxic/nutrient deprived conditions, drug resistance and metastasis variously downstream of Akt, SGK and PKC [3, 16, 17]. Finally, a number of functional cancer promoting mutations or amplifications have been identified in the mTORC2 components Sin1 and Rictor reinforcing distinct mTORC1 independent roles in cancer [18].

mTORC2 specific inhibitors remain to be described and in their absence alternative means are required to specifically assess this pathway as an independent target [19]. We previously identified Sin1 as a direct binding partner of the AGC kinases Akt and PKC and mapped binding to the CRIM (Conserved Region In Middle) domain. We went on to demonstrate that incorporation of AGC binding deficient mutants of Sin1 into endogenous mTORC2 uncoupled mTORC2 from these target effectors and influenced cellular growth in 3D. Here we have exploited this novel inducible dominant negative Sin1 strategy to uncouple mTORC2 signalling in a DLD1 colon cancer model. This reveals that suppression of mTORC2 alone is capable of restricting tumour development and further supports the rationale for developing mTORC2 specific drugs.

## RESULTS

### Sin1 truncation mutants incorporate into endogenous mTORC2 in DLD1 cells and suppress Akt phosphorylation

**Figure 1:**
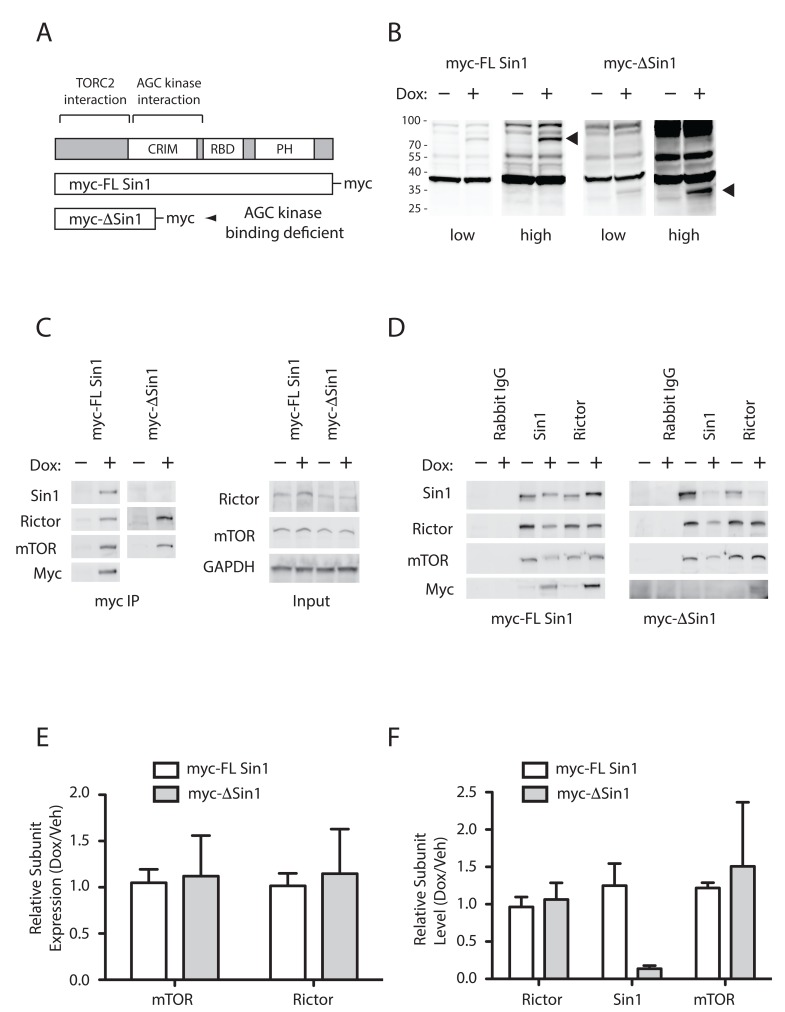
Truncated Sin1 displaces endogenous Sin1 from mTORC2 in DLD1 colon cancer cells **A.** Schematic indicating the domain structure of Sin1 and the constructs used to displace endogenous Sin1 from mTORC2. **B.** Expression of myc tagged Sin1 constructs can be detected only after induction with Doxycycline (Dox). Cells were treated with 100nM of doxycycline (+) for 72 hours and expressed proteins were detected by immunoblot of whole cell lysates with anti-myc (9E10) antibodies. **C.** and **D.** Sin1 constructs incorporate into mTORC2 and displace endogenous Sin1. Constructs were induced for 72 hours prior to immune precipitation. (C) mTORC2 subunits, mTOR and Rictor, only appear in myc immunoprecipitates after induction with doxycycline (Left panels); myc-∆Sin1 cannot be directly detected in precipitates due to secondary antibody cross reaction with precipitating IgG. Right panels indicate unchanging expression levels of Rictor and mTOR in immune precipitation input lysates, which is further quantified from 3 independent experiments **E.** Endogenous Sin1 and Rictor immunoprecipitates demonstrate displacement of endogenous Sin1 from mTORC2. Following induction, band shifted myc-tagged FL Sin1 can be detected in Sin1 and Rictor precipitates (Left panels). Truncated ∆Sin1 can be detected in Rictor, but not Sin1, immunoprecipitates as the Sin1 antibody epitope is deleted from ∆Sin1. **F.** Quantification of Sin1 levels detected in Rictor immunoprecipitates indicates the level of endogenous mTORC2 disruption following Sin1 construct induction (data are mean +/- S.D; *n* = 3). Myc-∆Sin1 displaces >80% of endogenous Sin1 while levels of myc-FL Sin1 associated with Rictor are comparable with endogenous Sin1 levels.

We previously identified that Sin1 interacts directly with AGC client kinases in yeast-2-hybrid and HEK293 pull-down assays, and mapped the interaction to the CRIM domain of Sin1 [21]. In this previous study, inducible expression of Sin1 mutants, which retain the C-terminal mTORC2 binding domain, but lack an intact CRIM domain, incorporate into the endogenous mTORC2 complex and inhibit hydrophobic motif targeting of both PKC and Akt in HEK293 cells. In order to examine a role in tumour growth we developed DLD1 colon cancer cell lines expressing doxycycline inducible myc-tagged full-length or truncated Sin1, which retains the mTORC2 binding domain (∆Sin1 - residues 1-192; Figure 1A). ∆Sin1 was selected from our panel of Sin1 constructs as it was the most efficient at uncoupling mTORC2 from both Akt and PKC phosphorylation in HEK293 cells [21]. This likely reflects the deletion of both the AGC recruiting CRIM domain and the membrane targeting PH domain [21, 22]. Myc-Sin1 constructs were introduced into FRT-DLD1 cells (courtesy of Stephen Taylor) using the FRT-TRex™ system (Invitrogen). Induction with doxycycline induced low-level expression of the constructs at the expected sizes (Figure 1B). Immune precipitation of the myc-tagged Sin1 co-purified both Rictor and mTOR confirming incorporation into the endogenous mTORC2 complex (Figure 1C). Myc-FL-Sin1 can also be detected using either Sin1 or myc (9E10) antibodies. Detection of the truncated ∆Sin1 in myc immunoprecipitates was not possible as the Sin1 epitope is absent and background from precipitating IgG obscured detection with the myc antibody. However, complex components were not purified in un-induced cells, confirming expression of the construct and specificity of the pull downs. Levels of mTOR and Rictor expression were unaltered by inducible Sin1 expression (Figure 1C and 1E); as previously described, detection of Sin1 in cell lysates is hampered by non-specific antibody cross reactivity [21]. We were unable to detect the localisation of the induced myc-tagged constructs by immunofluorescence, perhaps due to epitope masking or low levels of expression.

In order to examine and quantify the integrity of the mTORC2 complex, and the degree to which endogenous Sin1 has been displaced, we immunoprecipitated mTORC2 using either Rictor or Sin1 polyclonal antibodies (Figure 1D). Induction of ∆Sin1 expression resulted in reduced Rictor and mTOR in Sin1 immunoprecipitates. Endogenous Sin1 is also lost from Rictor immunoprecipitates, but levels of mTOR remain unchanged. Quantitation of mTORC2 complex components immunoprecipitated with Rictor before and after doxycycline induction across multiple experiments allows assessment of the penetrance of complex disruption (Figure 1F). ∆Sin1 expression resulted in a seven-fold reduction in levels of associated endogenous Sin1 (0.14 ± 0.04; average ± STD; *n* = 3) with no change in levels of associated mTOR. Together these data indicate that the ∆Sin1 construct incorporates into >80% of the endogenous mTORC2 complex without affecting the net expression levels of the complex. Levels of endogenous Sin1 immunoprecipitated by the Sin1 polyclonal were also reduced to the same degree (Relative Intensity 0.16 ± 0.14) indicating that displaced endogenous Sin1 is unstable and degraded [21]. Induction of myc-FL Sin1 had little effect on the total levels of Sin1 co-precipitated with Rictor (1.06 ± 0.2) although the endogenous doublet is entirely replaced by the band shifted myc-FL Sin1 (Figure 1D); as for ∆Sin1, endogenous Sin1 is displaced from Sin1 immunoprecipitates following myc-FL Sin1 incorporation into mTORC2. Assessment of mTOR and Rictor by immunofluorescence did not reveal any observable change in sub-cellular localisation in response to incorporation of either myc-Sin1 protein (Supplementary Figure S1).

**Figure 2:**
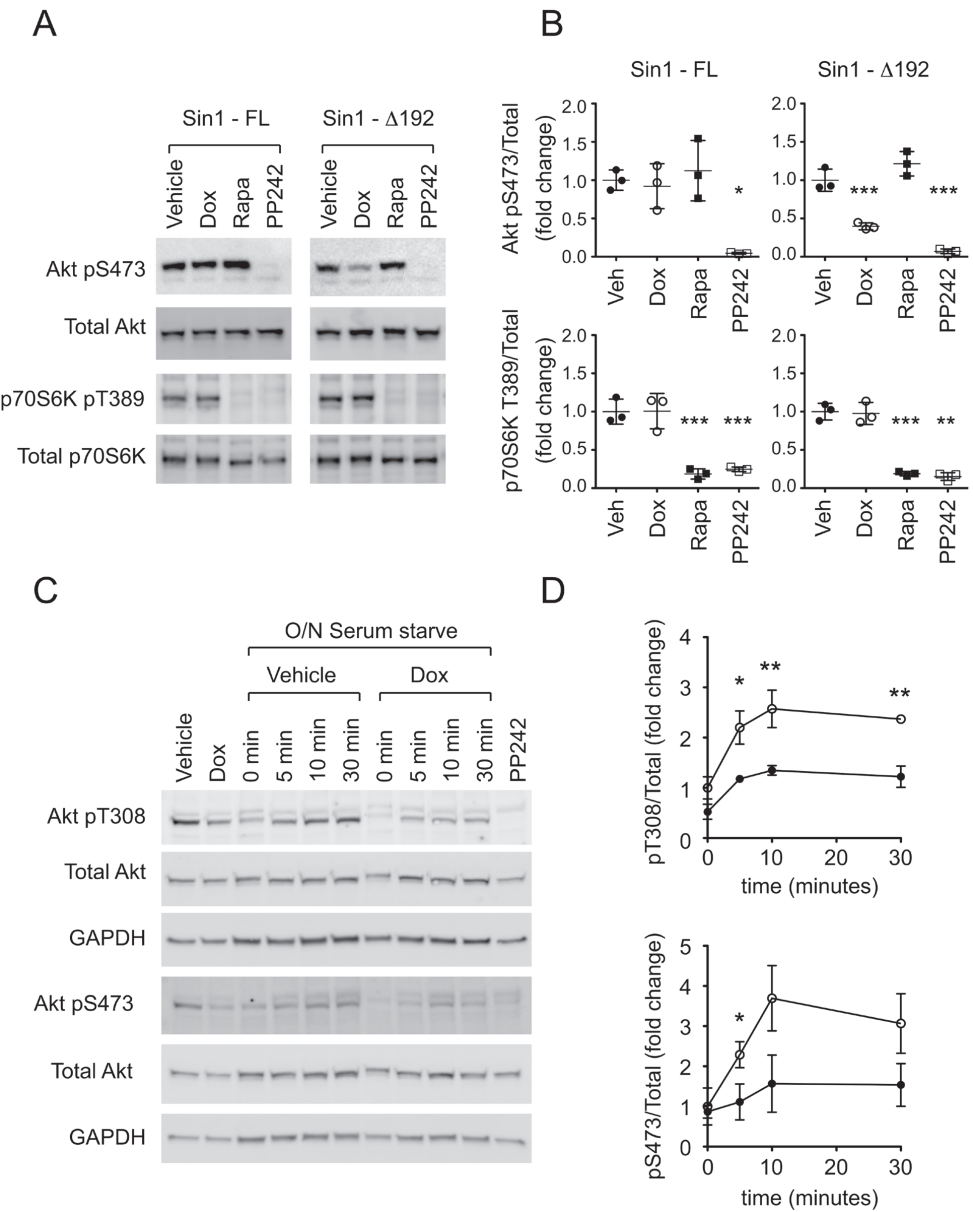
∆Sin1 expression suppresses Akt activation but not p70S6K activation in DLD1 cells **A.** Following 72 hours doxycycline (Dox) induction of Sin1 constructs, or 30 minute incubation with 1µM PP242 or 100nM rapamycin (Rapa), cell lysates were probed with the indicated antibodies. **B.** Quantification from 3 independent experiments indicates that Sin1∆1-192 but not Sin1-FL significantly inhibits phosphorylation of the mTORC2 target Akt on S473 but not the mTORC1 targeted p70S6K on T389. Conversely rapamycin selectively inhibits T389 phosphorylation while PP242 inhibits both. **C.** and **D.** Cells were serum starved (0.5% Serum) overnight (O/N)prior to stimulation with 10% Serum for the times indicated. Phosphorylation of Akt on pT308 and pS473 were assessed relative to total Akt. GAPDH indicates protein loading. Quantification represents mean +/- S.D (*n* = 3). Statistical significance was assessed by 1-way (B) or 2-way (D) ANOVA and Bonferroni post hoc tests; *p < 0.05; **p < 0.01; ***p < 0.001.

Consistent with our previous findings in HEK293 cells, ∆Sin1 expression in DLD1 cells suppressed Akt Ser473 phosphorylation but had no effect on phosphorylation of the mTORC1 target p70S6K Thr389 (Figure 2A and 2B). In contrast, inducible expression of full-length myc-Sin1 affected neither Akt nor p70S6K (Figure 2B). Rapamycin and the mTOR catalytic inhibitor, PP242, were used to confirm the respective targeting of p70S6K and Akt by mTORC1 and mTORC2 pharmacologically. To assess acute stimulation of Akt phosphorylation, serum starved DLD1 cells were stimulated with 10% serum. Serum induced Akt phosphorylation on both Ser473 and the PDK1 targeted activation loop (Thr308) w significantly inhibited by ∆Sin1 expression (Figure 2C and 2D). This likely reflects the combination of direct suppression of mTORC2 dependent S473 phosphorylation and reduced stability of activation loop phosphorylation in the absence of Ser473 phosphorylation.

Together these data demonstrate that inducible expression of Sin1 constructs can be used to modulate mTORC2 complex functionality while maintaining complex integrity. In contrast, Sin1 or Rictor ablation results in complex disruption with unknown adaptive consequences [10, 23].

### Suppression of mTORC2 activity blocks DLD1 xenograft tumour growth

**Figure 3:**
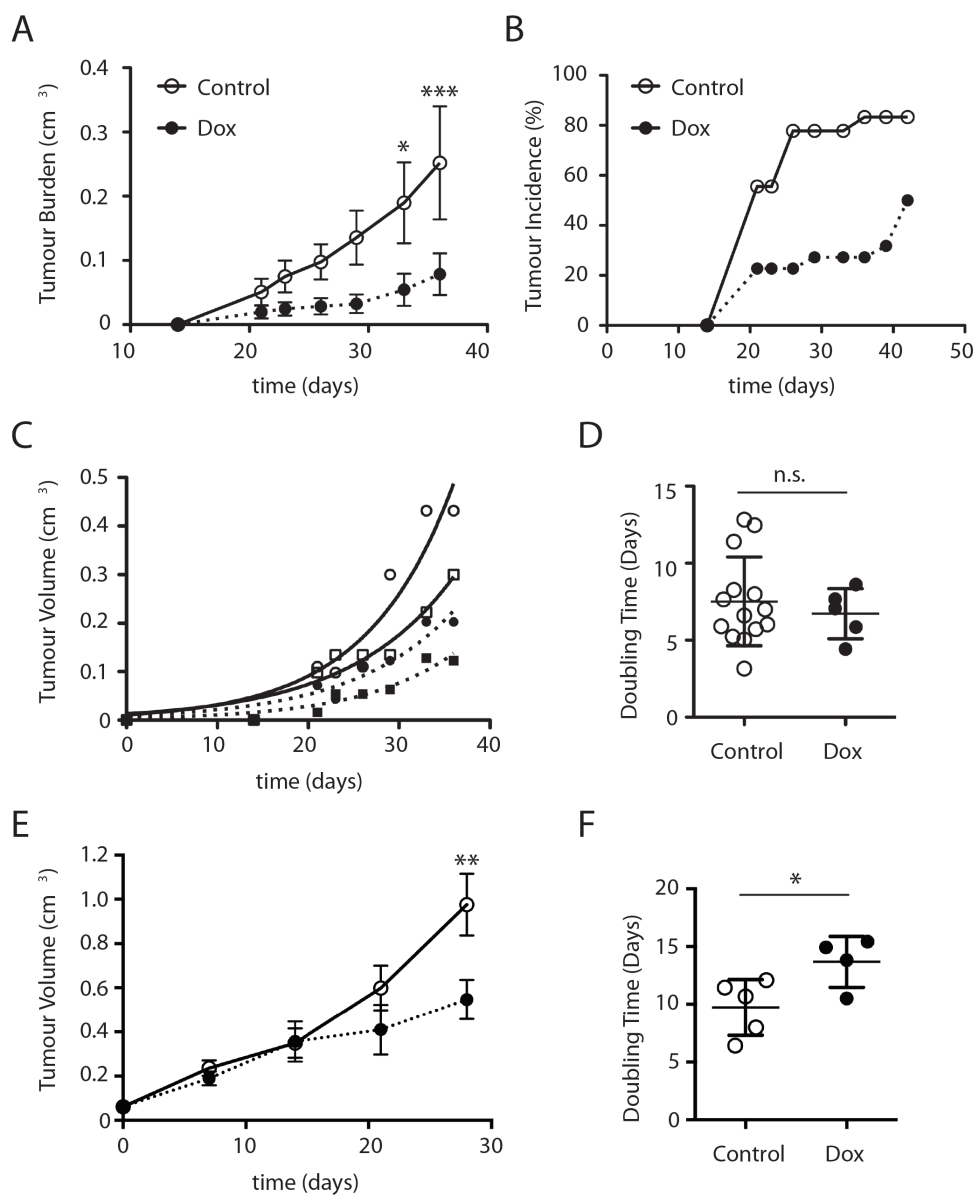
∆Sin1 expression suppresses DLD1 subcutaneous tumour growth in NOD/SCID mice Mice were inoculated with 10^6^ DLD1 cells on both flanks and assigned randomly to control or doxycycline (Dox) groups. Tumour burden **A.** and incidence **B.** per inoculation were assessed 3 times weekly. Data are mean +/- S.E.M. Statistical significance was assessed by ANOVA and Bonferroni post hoc test; **p < 0.01; ***p < 0.001. **C.** Examples of individual tumour growth rates; curves indicate exponential growth curve best fit to estimate growth rates. **D.** Growth rate of tumours does not differ significantly between control and doxycycline cohorts. Data are mean +/- STD **E.** Mice were inoculated on a single flank with DLD1 cells. Once tumours had reached a volume of 50mm^3^ mice were randomised into vehicle and Dox treated groups. Times indicated are post-1st treatment and statistical analysis was conducted as for A. This data is derived from a single cohort of animals. **F.** Growth rates for individual tumours from (E) are illustrated (student's t-test; *p < 0.05.

In order to assess a role for mTORC2 in tumour growth we conducted subcutaneous xenograft studies in NOD/SCID mice. Mice were injected with DLD1 ∆Sin1 tumour cells on both flanks and mice were randomly assigned to two groups: control and doxycycline. For doxycycline treatment, to induce ∆Sin1 expression, drinking water was supplemented with 1% sucrose (w/v) and 2mg/ml doxycycline; control mice were maintained on 1% sucrose alone as a vehicle control. Tumour growth was monitored three times weekly by caliper measurements and the experiment was terminated before tumour burden reached the maximum permitted volume. Doxycycline treatment significantly inhibited tumour incidence and average tumour burden (Figure 3A and 3B). Interestingly however, when we compared the growth rate of established individual tumours within the doxycycline group, doubling times were similar to those in the untreated group. Thus, despite a decrease in average tumour burden and incidence, tumours were able to grow at control rates once established.

To mimic therapeutic targeting we also assessed the effect of ∆Sin1 induction on the growth of established tumours (Figure 3E and 3F). Mice were injected with DLD1 ∆Sin1 tumour cells and assigned randomly to control or doxycycline groups when tumours reached 50mm^3^. Average tumour growth was modestly reduced by ∆Sin1 induction and individual tumour growth rate was also suppressed. In contrast to xenograft growth, ∆Sin1 expression did not affect growth of DLD1 cells under standard 2D cell culture conditions as previously described for HEK293 cells (data not shown). This concurs with the observation that Sin1 (unlike Rictor) knockout fibroblasts do not show a growth deficit in cell culture [10, 24]. We conclude that inducible suppression of mTORC2 function *in vivo* can suppress mTORC2 target phosphorylation and impede tumour development.

### Inhibition of tumour growth correlates with the degree of Akt suppression *in vivo*

**Figure 4:**
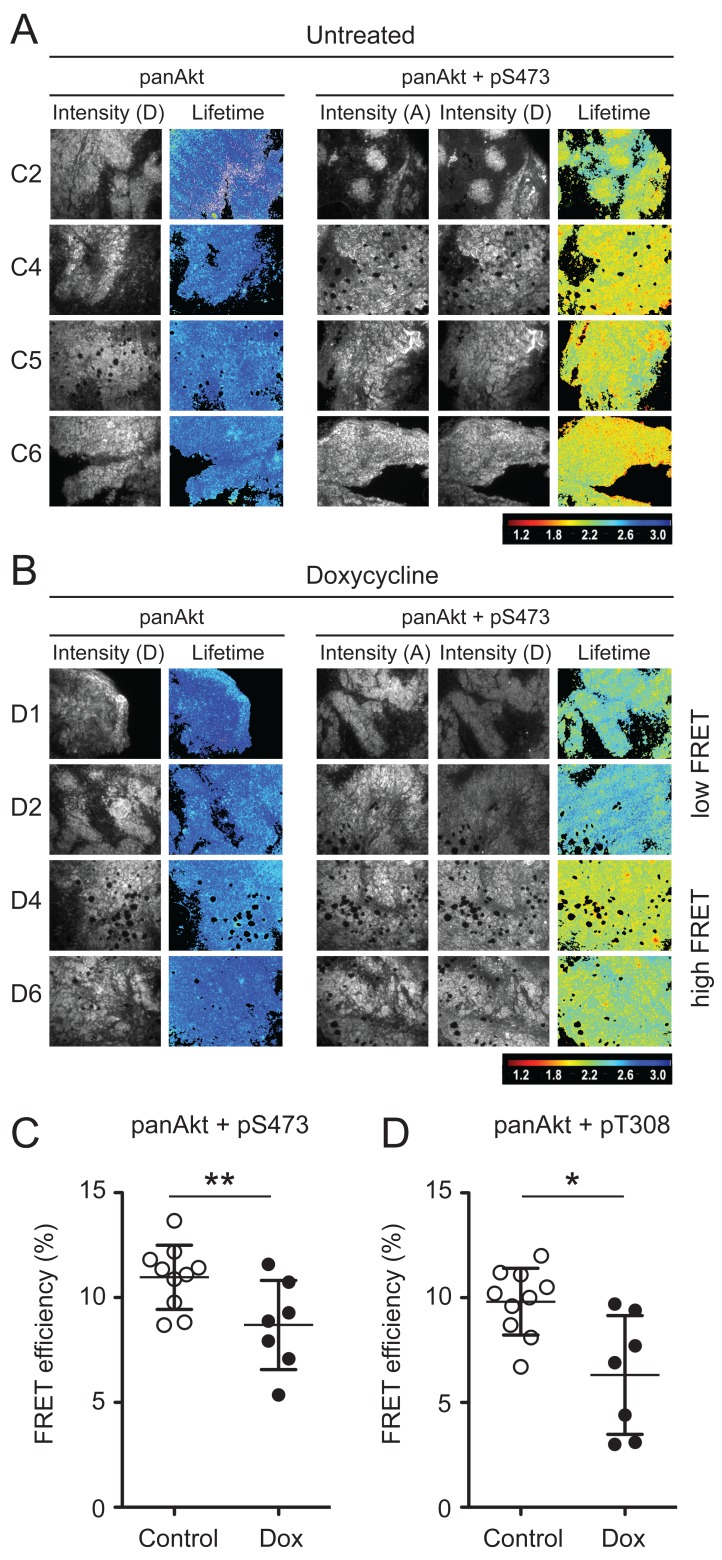
Time resolved amplified FRET indicates significant suppression of Akt activation following ∆Sin1 induction Example fluorescence intensity images and donor lifetime maps of tumours from four independent control (**A)** and doxycycline (**B)** treated mice. Tumours were stained for either panAkt (Donor; D) alone (Left panels) or panAkt and pS473 (Acceptor; A) together (Right panels). In the doxycycline cohort, representative examples of tumours showing high and low FRET efficiency are shown. **C.** and **D.** Multiple regions (*n* = 3-9) were quantified for each tumour and average FRET efficiency compared for control and doxycycline (Dox) cohorts; both pT308 and pS473 were significantly suppressed in the doxycycline cohort (student’s *t*-test; *p < 0.05; **p < 0.01) and results are the mean +/- S.D. FRET efficiency for individual tumour regions are presented in Supplementary Figure 2.

**Figure 5:**
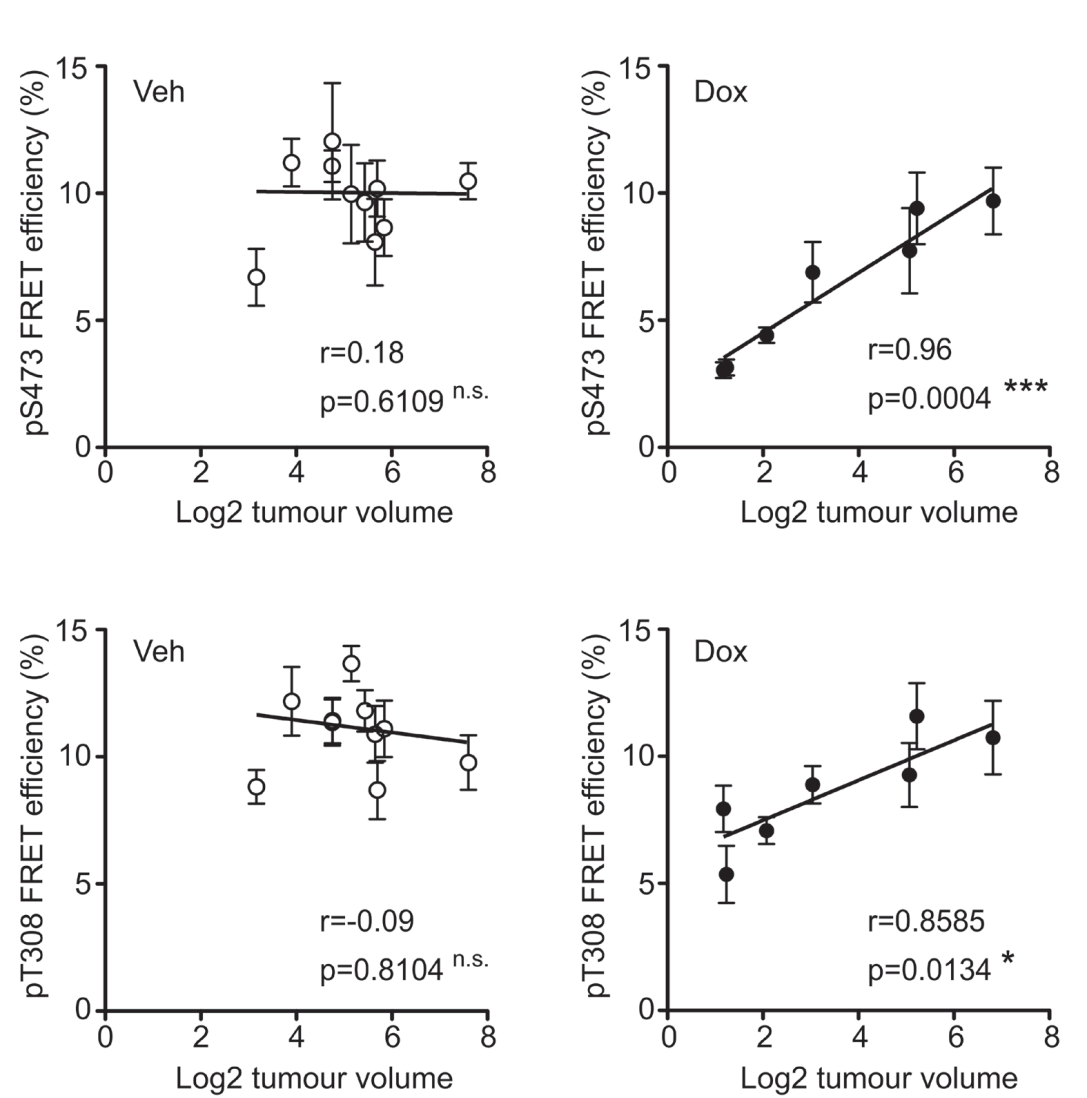
Tumour growth inhibition correlates with Akt suppression Each point indicates a single tumour and error bars indicate the mean +/- S.E.M. of quantified tumour regions. For both Akt pS473 (top graphs) and pT308 (bottom graphs) phosphorylation significantly correlates with tumour volume in the doxycycline (Dox) tumours (right graphs) but not the control tumours (vehicle treated; Veh) (left graphs). The Pearson correlation coefficient (r) and statistical significance for each condition is indicated.

To determine the whether ∆Sin1 was able to target mTORC2 function *in vivo* we conducted a time resolved amplified FRET (A-FRET) analysis of Akt activation status in tumour sections [20]. Here, we used an antibody directed against total Akt (mouse mAb) paired with either phospho-Akt Ser473 or phospho-Akt Thr308 (rabbit mAb). Total Akt was detected by Anti-mouse Fab-ATTO488 and pAkt (Ser473, Thr308) were detected by an Anti-rabbit Fab-HRP secondary which was further detected by using Alexa-594-TSA assay. FRET efficiency was assessed by monitoring the reduction in the fluorescence lifetime of the donor fluorophore (ATTO488) across tumour sections; donor lifetime maps in the presence of the acceptor provide a direct measure of Akt phosphorylation (Figure 4A and 4B). FRET efficiency was quantified across a cohort of control and doxycycline tumours collected at a single time point. For each tumour, multi-region comparisons allow assessment of variation in the levels of Akt phosphorylation both within and between tumours. Both Ser473 and Thr308 phosphorylation were on average significantly lower in tumours from doxycycline treated mice compared with untreated mice (Figure 4C, 4D and Supplementary Figure 2). However, a number of tumours in the doxycycline group demonstrated FRET efficiencies comparable with tumours from the control groups and significantly, levels of Akt activation were found to correlate with tumour size (Figure 5). This implies that disruption of mTORC2 function can impede tumour growth but that compensatory re-activation of downstream targets may provide an escape mechanism allowing tumours to re-establish growth rates comparable with control tumours.

## DISCUSSION

The first generation of mTOR targeting drugs stem from the eponymous allosteric inhibitor of mTORC1, rapamycin. Rapamycin and its analogues (rapalogues) have been approved for the treatment of a number of malignancies, including Renal Cell Carcinoma, HER2 negative breast cancer and various neuroendocrine tumours [2, 3]. Despite some success, rapalogues exhibit a number of undesirable biochemical properties. Firstly, they only partially inhibit mTORC1 signalling in a substrate specific manner, as the key target 4E-BP1 escapes complete inhibition. Furthermore, mTORC2 signalling to pro-growth pathways, including Akt, remains intact and is in fact amplified as inhibition of mTORC1 releases negative feedback control of the PI3kinase/mTORC2/Akt pathway. Finally, as rapalogues are generally cytostatic, prolonged treatment regimes may be required with unfavourable toxicity profiles at effective doses.

Second generation active site directed mTOR inhibitors, that target both mTORC1 and mTORC2, demonstrate significantly improved anti-cancer activity in preclinical models when compared with rapalogues [25-30]; a variety of these mTOR inhibitors are now being explored in the clinic with mixed results [12, 31-34]. While targeting both arms of mTOR signalling shows promise, strategies for selective targeting of mTORC2 alone could be beneficial or indeed a preferred strategy in some diseases. In particular, PTEN/PI3K mutant tumours, and tumours where activating mutations or amplifications in mTORC2 components have been identified may respond to more selective interventions with reduced liabilities. Additionally, there are situations where targeting mTORC1 may be ill advised. For example, under nutrient deprived conditions, inhibition of mTORC1 has been shown to promote tumour growth by enhancing pro-survival autophagy and cellular use of proteins as an energy source [13, 14]. Undesired consequences associated with mTORC1 inhibition, such as immune suppression, might also be avoided and this has particular relevance to combination studies in this era of immune-oncology.

In order to model the effects of targeting mTORC2 alone, strategies which perturb the function of the mTORC2 specific subunits Sin1 or Rictor are necessary. Rictor and Sin1 null cells from knockout mice have thus been instrumental in defining mTORC2 complex regulation and targets. Deletion of either Sin1 or Rictor results in complete loss of the mTORC2 complex [10, 35]. As an alternative strategy, we developed an inducible system for disrupting recruitment of mTORC2 client kinases to the endogenous complex. Here, truncated Sin1, which cannot bind to AGC kinases, replaces endogenous Sin1 without altering the net levels of assembled cellular mTORC2 complex. In a previous study we found that truncations, which delete both the CRIM domain (responsible for AGC kinase recruitment [36]) and the C-terminal PH domain, were the most effective at supressing Akt phosphorylation. The Sin1 PH domain has recently been shown to mediate direct inhibition of the mTOR kinase domain, analogous to the inhibition of Akt by its own C-terminal PH domain [22]. Binding of the Sin1 PH domain to PtdIns(3,4,5)P_3_ downstream of PI3 kinase both releases inhibition and co-localises mTORC2 with its substrate Akt. Interestingly, in a previous study, we found that incorporation of PH domain disrupted Sin1 into mTORC2 did not significantly modify basal Akt phosphorylation in HEK293 cells [21]. This somewhat counterintuitive observation likely reflects the ability of Sin1 to directly interact with Akt combined with the dis-inhibition of mTOR by PH domain deleted Sin1. These observations are consistent with the model wherein membrane associated, PDK1 phosphorylated Akt is recruited to mTORC2 via Sin1, promoting phosphorylation of Sin1, activation of mTORC2, triggering S473 phosphorylation on Akt [21, 37].

Despite regulation through a genetic mechanism, we have generated a system where we can inducibly ablate approximately 90% of cellular TORC2 activity following doxycycline administration; this pharmacomimetic system provides unique insight into the potential *in vivo* response to mTORC2 blockade. Encouragingly, suppression of mTORC2 significantly impeded tumour development in our xenograft model. Two-site amplified FRET analysis revealed target suppression *in vivo* with significant reduction in phosphorylation of Akt on both mTORC2 targeted Ser473 and PDK1 targeted Thr308 (a weaker inhibitory effect was observed on this latter site). Associated with the direct effect of mTORC2 inhibition, loss of Thr308 was also observed in cell culture. This likely results from reduced stability of Thr308 phosphorylation in the absence of Ser473 mediated kinase domain stabilisation. An alternative mechanism, where disruption of PIF pocket dependent recruitment of PDK1 in the absence of Akt Ser473 phosphorylation is unlikely to explain Thr308 reduction, as blocking this interaction is not sufficient to block Akt activation by PDK1 [38]. Interestingly, once tumours became established growth rates were comparable in both control and mTORC2 supressed groups and the degree of pAkt suppression correlated with inhibition of tumour growth. The high level of Akt phosphorylation in more rapidly growing tumours may be explained through multiple mechanisms. Firstly, Akt Ser473 may be reacquired through adaptive changes, in the absence of mTORC2; retention of Akt Ser473 has been observed in tissue-specific Rictor knockouts [39], potentially involving DNA-PK, which can also mediate this phosphorylation [40]. Alternatively, up regulation of the less efficient mTORC2 complex or expansion of ∆Sin1 null subpopulations of DLD1 cells may account for the escape. Clearly the mechanism underlying mTORC2 blockade escape warrants further investigation in addition to exploring combination regimes, however the significance of the current findings is evident - selective inhibition of mTORC2 elicits a tumour suppressive response.

## MATERIALS AND METHODS

### Cell culture and tumour inoculation

FRT-DLD1 cells were cultured in DMEM, 10% foetal bovine serum, penicillin (50 units/ml) and streptomycin (0.05 mg/ml) in 10% CO_2_. Tetracycline-inducible Sin1 lines were generated using the pcDNA5.0 FRT T-Rex™ system (Invitrogen) according to the manufacturer’s instructions. Sin1 expression was induced with 100 ng/ml tetracycline. Animal studies were compliant with UK Home Office regulations and carried out under license PPL 70/8066. Female NOD/SCID mice were inoculated subcutaneously on the hind flank with 1x10^6^ DLD1 cells before being assigned randomly to control and doxycycline cohorts. Drinking water was supplemented with 1 % sucrose (w/v) and 2mg/ml doxycycline. Tumour growth was monitored by caliper measurements twice weekly and volume calculated as (w^2^ x l)/2. Excised tumours were fixed in formalin and paraffin embedded for pathology and FRET analysis.

### Antibodies and reagents

Mouse anti-Akt/PKB (A-FRET) mAb (SKB1) was from Millipore (#: 05-591), anti-pAkt (Thr308) (D25E6) rabbit (#: 13038S), anti-pAkt (Ser473) (D9E) rabbit (#: 4060S), and anti-Akt (immunoblot) mAb (#:40D4) were obtained from Cell Signaling Technology. Affinity-purified F(ab’)2 fragments Perox-Apure, Fab-Frag Anti-Rabbit -HRP (#: 711-036-152) were purchased from Jackson ImmunoResearch, Suffolk, UK. Peroxidase Suppressor (#35000) and Tyramide Signal Amplification (TSA) kit with Alexa Fluor 594 tyramide (#: T-20925) was purchased from Thermo Fisher Scientific, UK. ATTO488 NHS-ester dye (#: 41698-1MG-F) from Sigma. Sin1 (NB110-40424), Rictor (NB100-612) and mTOR (NBP-19855) polyclonal antibodies were purchased from Novus Biologicals. Anti-myc (9E10) was prepared by the In-House antibody facility. Sin1 mAb was from R&D Systems (MAB8168).

### Immunoprecipitation and immunoblotting

For immunoprecipitation of mTORC2, cells were lysed in 0.3% CHAPS, 40 mM Tris pH 7.4, 120 mM NaCl, 1 mM EDTA, 50 mM NaF, 10 mM Pyrophosphate, 10 mM β-glycerophosphate, 2 mM Na orthovanadate supplemented with cOmplete™ protease inhibitor cocktail (Roche). 2 µg of antibody was conjugated to protein G DynaBeads (Invitrogen) and incubated overnight with 1-2mg centrifuged lysate and washed four times with lysis buffer. Immunoprecipitates or cell lysates were resolved by SDS-PAGE, transferred to nitrocellulose and incubated with designated antibodies overnight. Bands were visualised and quantified using an AI600 imaging system (GE Healthcare), using HRP conjugated secondary antibodies and enhanced chemiluminescence.

### Conjugation of Fab-fragment with ATTO488-NHS dye

ATTO488-NHS conjugation to the anti-mouse-specific Fab secondary antibody (1mg/ml) was performed. Briefly, the ATTO488-NHS-ester dye was reconstituted in anhydrous DMF to a concentration of 1mg/ml and added (125µg of dye/1mg of protein) to the Fab fragment antibody solution. The reaction mixture was incubated with constant agitation at room temperature for 1 hour. Purification of labelled antibodies was performed using pre-equilibrated (PBS, pH 7.2) gravity flow PD-10 dye removal columns. The dye: protein ratio required for these experiments should not exceed a 4:1 ratio.

### Quantification of pAkt (pT308/p473) in mouse FFPE tumour by time resolved two-site amplified FRET (A-FRET) assay

For the acquisition of donor (ATTO488) fluorescent lifetime an automated multiple frequency lifetime imaging microscope (Lambert Instruments) was used. Two identical tumour sections (4 μm) were de-waxed, rehydrated and subjected to heat antigen retrieval by microwaving in Tris-EDTA (pH 9.0) buffer, for 10 minutes. Sections were then incubated in freshly prepared sodium borohydride (1mg/ml in PBS) buffer for 10min at RT, followed by blocking with 1% BSA/PBS. Tissue sections were incubated with peroxidase suppressor for 15min. For the A-FRET assay, the first slide was incubated with mouse anti-panAkt (1:50), and the second slide with mouse anti-panAkt (1:50) plus rabbit anti-pT308 (1:200) primary antibodies, overnight at 4°C. The first slide was further immunolabeled with (donor fluorophore) ATTO488-conjugated anti-mouse Fab fragment secondary antibody (20µg/ml). The second slide was immunolabeled with ATTO488-conjugated anti-mouse Fab fragment (20µg/ml) and HRP-conjugated anti-rabbit Fab fragment secondary antibody (10µg/ml), which was detected by using (acceptor fluorophore) Alexa-594-TSA assay. These slides were mounted with ProLong^®^ Gold anti-fade. The donor lifetimes of ATTO488 were determined from at least 3 regions from each section with elevated donor intensity. Lifetime data was analysed using a purpose-built algorithm and the FRET efficiency was calculated using the following equation: E_f_ = 1-(*t*DA/*t*D)*100%; where *t*D is donor lifetime and *t*DA is donor plus acceptor lifetime [20]. After the FRET quantification, the above slides were scanned under a confocal microscope to assess the co-localization of panAkt and pT308/p473.

## SUPPLEMENTARY MATERIALS FIGURES



## Abbreviations

mTOR: Mammalian target of rapamycin
mTORC2: Mammalian target of rapamycin complex 2
PKC: protein kinase C
CRIM: Conserved Region In Middle
STD: Standard Deviation
