# Supplementary material for: Uncoupling TORC2 from AGC kinases inhibits tumour growth
Source: Oncotarget. 2017 Aug 9;8(49):84685–96. doi: 10.18632/oncotarget.20086 (PMC5689566; doi:10.18632/oncotarget.20086)
Supplement: Supplementary file 1 [file oncotarget-08-84685-s001.pdf]

Uncoupling TORC2 from AGC kinases inhibits tumour growth

SUPPLEMENTARY MATERIALS

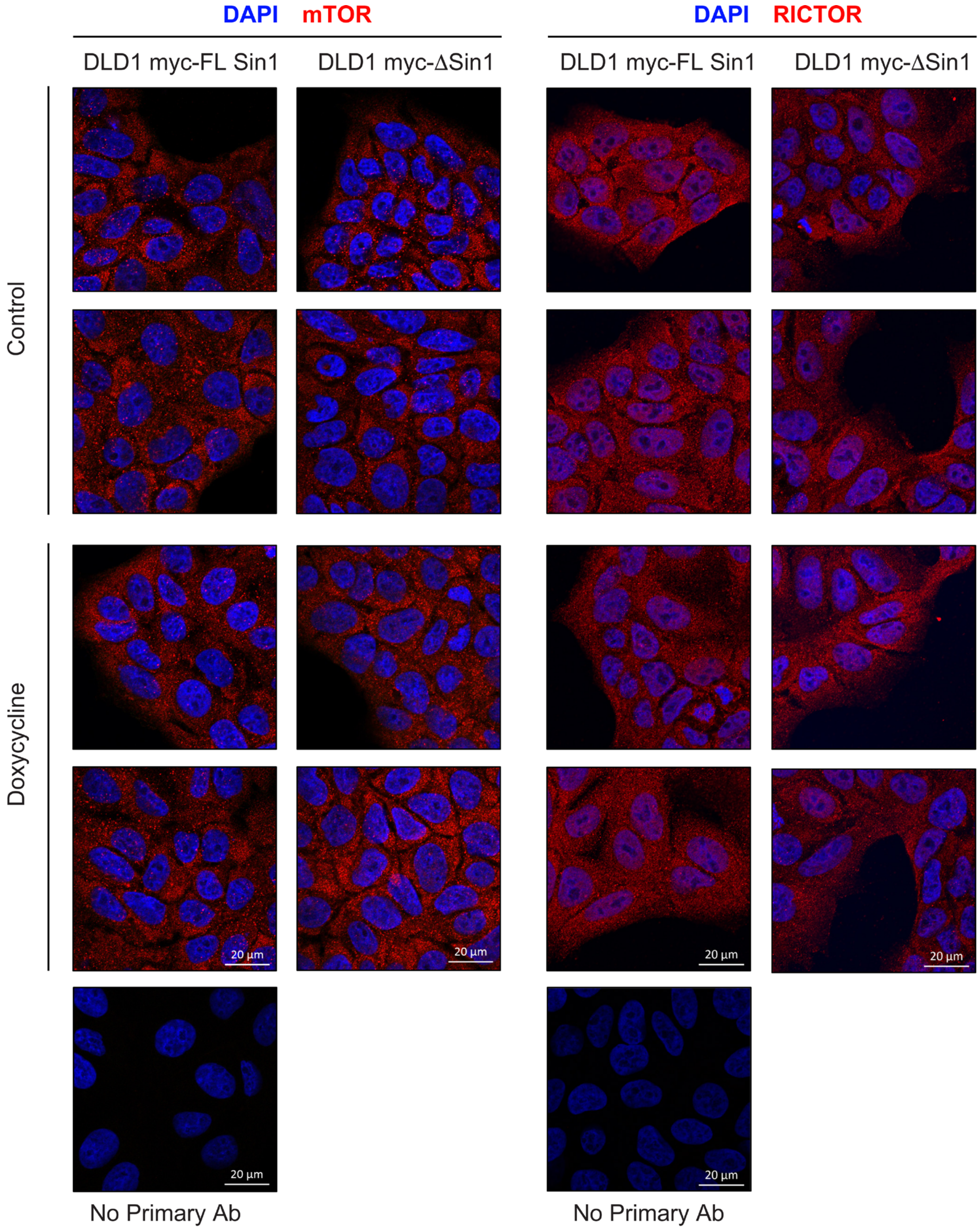

Supplementary Figure S1. mTOR and Rictor immunostaining of inducible Sin1 contruct expressing DLD1 cells. myc-FL Sin1 or myc ΔSin1 DLD1 cells were seeded onto glass coverslips in 24-well plates and treated with doxycycline or vehicle control for 3 days as indicated. Cells were fixed in 4% PFA, washed, and permeabilised in PBS containing 0.1% (v/v) Triton-X100, 2% (w/v) BSA for 15 minutes. Cells were incubated with anti-mTOR or anti-Rictor polyclonal antibody followed by incubation with an Alexa Fluor 555-conjugated secondary antibody and DAPI. Images were captured on a Zeiss LSM 710 confocal microscope.

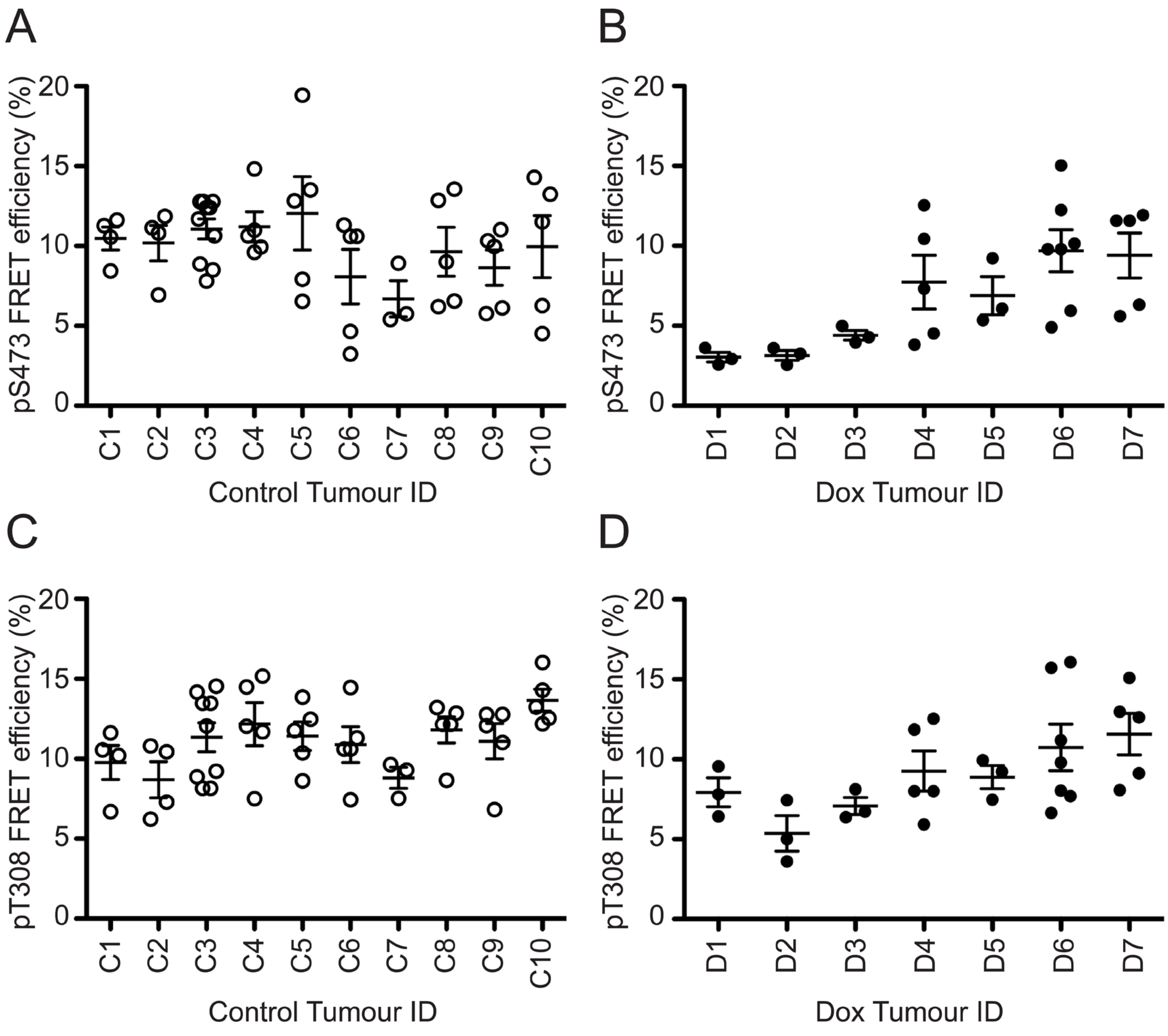

Supplementary Figure S2. FRET efficiency measurements for individual tumour regions. Time resolved amplified FRET was used to assess Akt phosphorylation status for multiple randomly selected regions (n=3-9) for each tumour. Average FRET efficiency compared for control and doxycycline (Dox) cohorts are presented in Figure 4 of the manuscript. Panels A and B show individual region measurements for pAkt S473 for control and doxycycline (Dox) tumours respectively. Panels C and D show individual region measurements for pAkt S308. Mean with SEM are indicated for each tumour.
